# Impact of the large-scale deployment of artemether/lumefantrine on the malaria disease burden in Africa: case studies of South Africa, Zambia and Ethiopia

**DOI:** 10.1186/1475-2875-8-S1-S8

**Published:** 2009-10-12

**Authors:** Karen I Barnes, Pascalina Chanda, Gebre Ab Barnabas

**Affiliations:** 1 Division of Clinical Pharmacology, Department of Medicine, University of Cape Town Faculty of Health Sciences, Anzio Road, Observatory, 7925, South Africa; 2 Ministry of Health, Department of Public Health and Research, P.O. Box 30205, Lusaka, Zambia; 3 Tigray Health Bureau, Mekelle, Tigray, Ethiopia

## Abstract

Malaria is one of the most significant causes of morbidity and mortality worldwide. Every year, nearly one million deaths result from malaria infection. Malaria can be controlled in endemic countries by using artemisinin-based combination therapy (ACT) in combination with indoor residual spraying (IRS) and insecticide-treated nets (ITNs). At least 40 malaria-endemic countries in sub-Saharan Africa now recommend the use of ACT as first-line treatment for uncomplicated falciparum malaria as a cornerstone of their malaria case management. The scaling up of malaria control strategies in Zambia has dramatically reduced the burden of malaria. Zambia was the first African country to adopt artemether/lumefantrine (AL; Coartem^®^) as first-line therapy in national malaria treatment guidelines in 2002. Further, the vector control with IRS and ITNs was also scaled up. By 2008, the rates of in-patient malaria cases and deaths decreased by 61% and 66%, respectively, compared with the 2001-2002 reference period.

Treatment with AL as first-line therapy against a malaria epidemic in the KwaZulu-Natal province of South Africa, in combination with strengthening of vector control, caused the number of malaria-related outpatient cases and hospital admissions to each fall by 99% from 2001 to 2003, and malaria-related deaths decreased by 97% over the same period. A prospective study also showed that gametocyte development was prevented in all patients receiving AL. This reduction in malaria morbidity has been sustained over the past seven years.

AL was introduced as first-line anti-malarial treatment in 2004 in the Tigray region of Ethiopia. During a major malaria epidemic from May-October 2005, the district in which local community health workers were operating had half the rate of malaria-related deaths compared with the district in which AL was only available in state health facilities. Over the two-year study period, the community-based deployment of AL significantly lowered the risk of malaria-specific mortality by 37%. Additionally, the malaria parasite reservoir was three-fold lower in the intervention district than in the control district during the 2005 high-transmission season.

Artemisinin-based combination therapy has made a substantial contribution to reducing the burden of malaria in sub-Saharan Africa.

## Background

Malaria is a preventable and treatable disease, yet in endemic countries in Africa, the consequences of malaria can be catastrophic. It is thought that approximately half of the world's population is currently at risk from malaria (3.3 billion people) [1]. In 2006, there were an estimated 247 million clinical cases of malaria, 86% of which were in Africa [2]. Every year, nearly one million deaths result either directly or indirectly from malaria infection, most of them in children under five years of age [2]. This is equivalent to a child dying of malaria in Africa every 30 seconds [3].

In addition to the human burden of malaria, the economic cost is staggering. Malaria causes an average loss of 1.3% of annual economic growth in countries with intense transmission [1], and the estimated economic burden to African countries is 12 billion USD per year [4]. For a fraction of that sum, malaria could be controlled. Malaria can be prevented and managed in endemic countries by using a combination of three main strategies: indoor residual spraying (IRS), insecticide-treated nets (ITNs), and treatment with artemisinin-based combination therapy (ACT). ACT has the unique advantage of reducing malaria transmission (by decreasing gametocyte carriage) while curing the disease. Employing tools for malaria prevention as well as treatment can have a remarkable impact on the disease burden.

Artemether/lumefantrine (AL; Coartem^®^) is an ACT that offers PCR-corrected 28-day cure rates of >95% [5-13], if given in a six-dose regimen. AL meets the World Health Organization (WHO) pre-qualification criteria for efficacy, safety and quality and is the only ACT that has been approved by ICH stringent regulatory authorities [14]. A landmark private-public agreement between Novartis and WHO was unveiled in 2001, whereby Novartis agreed to make AL available without profit in malaria-endemic developing countries.

With an increasing number of countries deploying ACT, the next challenge is to measure the impact these drugs are having on malaria. Studies designed to measure the health impact of a particular intervention can be conducted at different levels of stringency, and a variety of end points can be used to measure the outcome e.g. all-cause mortality, malaria-specific mortality, and infection/gametocyte prevalence. A number of other factors require careful consideration when evaluating the credibility of results from trials designed to assess the impact of ACT; these include changing coverage of other strategic interventions (e.g. insecticide-treated nets and indoor residual spraying), and changing diagnostic practices (e.g. introduction of rapid diagnostic tests). To optimize such assessments, studies should collect information from multiple sites, standardize case definition, and collect data over a prolonged period of time. Here, we discuss the results of studies from South Africa, Zambia and Ethiopia that assessed the impact of AL on malaria morbidity and mortality.

## KwaZulu-Natal province, South Africa: malaria deaths fell by over 90%

**Figure 1:**
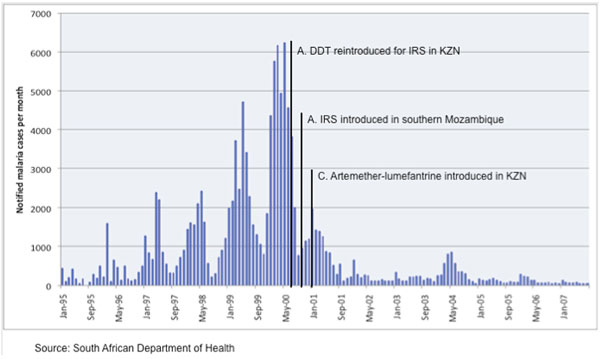
**Number of notified cases of confirmed malaria in KwaZulu-Natal by month **[15]. A indicates reintroduction of dichlorodiphenyltrichloroethane (DDT) for indoor residual spraying of traditional structures in KwaZulu-Natal; B indicates introduction of community-based indoor residual spraying in neighbouring Southern Mozambique; C indicates the implementation of artemether/lumefantrine as first-line therapy in KwaZulu-Natal.

**Figure 2:**
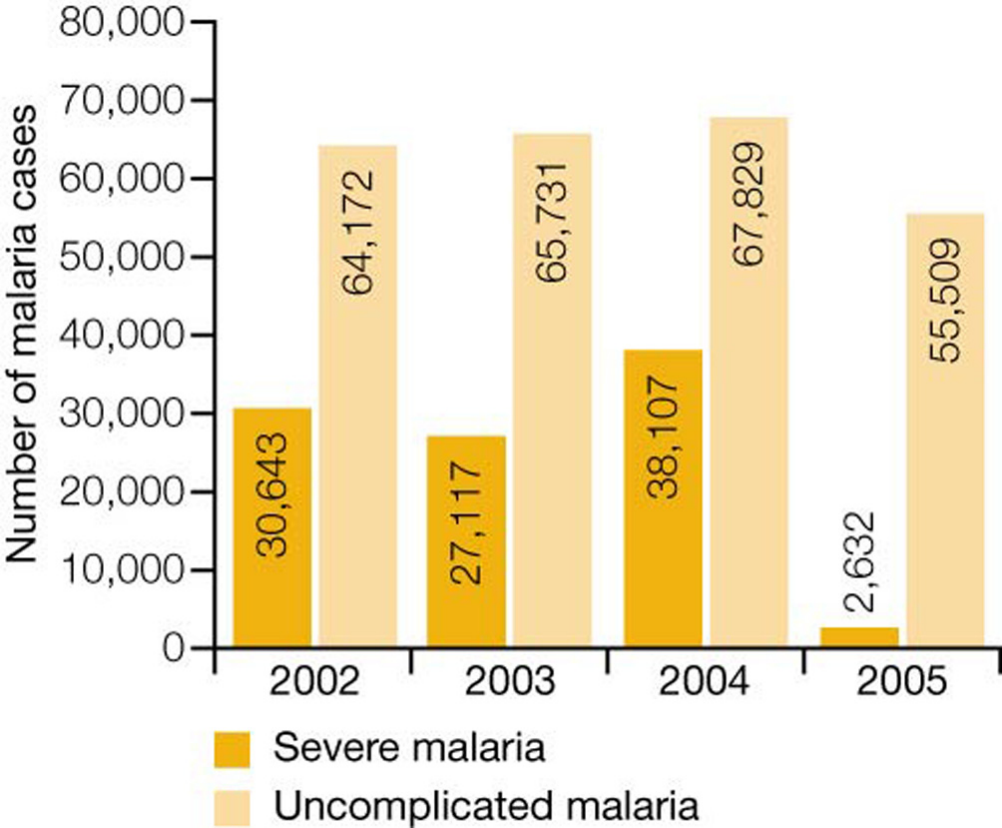
**Number of cases of severe and uncomplicated malaria in Zambia by year (from 18 cost-effectiveness study sites)**.

The first large-scale use of AL in Africa was in the KwaZulu-Natal province of South Africa. During 1995-2000, the area experienced a marked increase in falciparum malaria, fuelled by a rise in resistance to pyrethroids used for IRS and to the treatment recommended, sulfadoxine-pyrimethamine. Following rapid regulatory approval of AL, the drug was launched as first-line anti-malarial therapy in KwaZulu-Natal in January 2001. The introduction of AL, together with a bold regional programme for strengthening IRS in both KwaZulu-Natal and neighbouring southern Mozambique, had a dramatic effect. Malaria-related outpatient cases reduced by 85% in 2001 (Figure 1), and by 2003, the number of malaria-related outpatient cases and hospital admissions had each fallen by 99%, and malaria-related deaths had decreased by 97% [15]. This considerable reduction in the malaria burden has been sustained over the past 7 years (Figure 2). In a prospective study with 42 days follow-up, AL had a cure rate of 99% and prevented gametocyte development in all patients. Adherence to the six-dose AL regimen was reported as 96%, which is similar to the value (93%) reported in a recent study in Bangladesh [16].

A cost-effectiveness analysis in the KwaZulu-Natal region has demonstrated that although AL treatment is more expensive than SP, its superior cure rate and the reduction in malaria transmission account for significant cost savings [17]. Use of AL resulted in an overall reduction in total malaria expenditure and considerable cost-savings of US$201,065 in 2002 alone in the one rural hospital and its nine clinics studied, when compared with SP.

The change in anti-malarial treatment policy to AL in KwaZulu-Natal has saved a significant number of lives, by improving clinical and parasitological cure rates and reducing gametocyte carriage and thus malaria transmission.

## Zambia: first African country to deploy artemether/lumefantrine as first-line treatment

Zambia was the first African country with nationwide endemic malaria to adopt AL as first-line therapy according to national malaria treatment guidelines [18]. This is because the 14-day or 28-day failure rates with the anti-malarial treatment chloroquine in Zambia had exceeded 40% [19,20]. In 2003, seven pilot districts commenced treatment with AL [21], followed by 23 districts and then nationwide deployment in all 72 districts by December 2004. The AL was provided free of charge at all the public health centres countrywide. The proportion of children treated with AL increased from 10.7% in 2004 to 42% in 2006 [22]. The Zambian Ministry of Health and partners also took steps to increase vector control efforts using IRS and ITNs. Between 2003 and 2005, more than five million nets were distributed, while the number of structures sprayed increased from 69,774 in 2003 to 657,695 in 2007 [23].

Between 2001 and 2003, the number of malaria deaths declined from 9,369 to 9,178 (2% reduction; Table 1); however, after the scale-up of the malaria control efforts in 2003, the number of malaria-related deaths decreased from 9,178 to 6,484 between 2003 and 2006 (30% reduction) [24]. Surveillance data has shown that the rates of in-patient malaria cases and deaths in 2008 decreased by 61% and 66%, respectively, compared with the reference period (2001-2002) before the control efforts were accelerated [21]. Additionally, a cost-effectiveness analysis in Zambia has indicated that the health gains (as defined by treatment success) from every dollar spent are significantly greater if AL is used rather than SP [25]. Another important finding reported in the study was a 91-93% reduction in severe malaria cases at health facilities, as shown in Figure 2[25].

**Table 1:** Number of all-cause and malaria-related deaths in Zambia from 2001-2006 [24].

	**All age-groups (including under 5 years of age)**		
	
**Year**	**Total all-cause deaths**	**Total malaria attributed probable deaths**	**Total malaria as a proportion (%) of all deaths**
2001	35358	9369	26.50
2002	39482	9021	22.85
2003	39117	9178	23.46
2004	38466	8289	21.55
2005	38740	7737	19.97
2006	35541	6484	18.24

This investigation revealed no differences in data registration or the definition of severe malaria between the retrospective and prospective periods that could account for this reduction. Severe malaria cases as a proportion of uncomplicated malaria cases have declined even more drastically from 2004 to 2005, indicating that fewer cases than before are progressing to severe malaria [25].

Data from population-based malaria indicator surveys have confirmed the reduction in malaria morbidity being recorded at the health facility level. Malaria parasite rates from a nationally representative sample have reduced by more than 50% (22% in 2006 *versus *10% in 2008) in children under five years of age [26,27]. Other site specific data show that parasite prevalence in febrile patients has decreased from 83% in 2004 to less than 1% in 2008 [28]. Health facility data from the paediatric department of the Macha Hospital in Southern Zambia has demonstrated that malaria cases declined by more than 50% between 2003 and 2005 after the introduction of AL, even before vector control strategies were scaled up in the area [29]. Malaria experts in Zambia believe that AL has been a major factor in the reduction in malaria morbidity.

The implementation of effective treatment and large scale vector control have resulted in reductions in malaria morbidity and mortality in Zambia, as demonstrated by both facility and population surveys [23,26-29]. Maintaining these strategies is key as Zambia is expected to be one of the few countries to achieve the Roll Back Malaria (RBM) target of reducing malaria mortality by half by 2010.

## Providing diagnosis and treatment at a local level through community health workers: the Tigray project, Ethiopia

A two-year pilot project, commencing in 2005, was undertaken in Ethiopia to assess how training and equipping local community health workers with AL and rapid diagnostic tests (RDTs) could help to achieve effective management of malaria in rural areas. The project took place in Tigray, the most northern region of Ethiopia (~80,000 km^2^) that has a largely rural (81%) population of approximately 4.5 million. Overall, 56% of the population of Tigray live in areas in which malaria is endemic, and less than half of the population live within easy reach of a health centre [30]. AL was introduced as first-line anti-malarial treatment in 2004 in the Tigray region following a high 14-day treatment-failure rate with SP in Ethiopia (35.9% in 2003; [31]), with large-scale deployment of AL beginning in 2005.

Unusually for Africa, *Plasmodium falciparum *and *Plasmodium vivax *coexist in the Tigray population. Malaria transmission is seasonal and hypo-endemic, making the area vulnerable to epidemics because of the low levels of immunity within the population.

The Tigray project had two main objectives:

1. To assess the impact of community deployment of AL on malaria morbidity, hospital admissions, in-patient deaths, slide positivity rate, mortality and health services utilization.

2. To assess the feasibility and impact of phased introduction of community-based rapid diagnostic tests to confirm diagnosis of malaria before the administration of AL.

**Figure 3:**
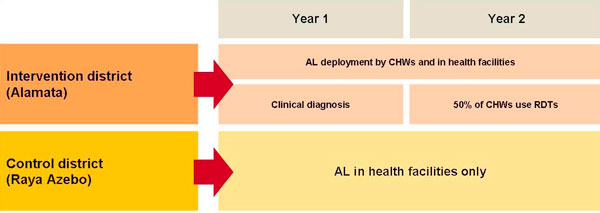
**The Tigray Project Study Design**.

To examine the impact of community deployment of AL, the study area was divided into two districts (Figure 3). In the control district (Raya Azebo), standard malaria case-management continued, with AL being prescribed at health facilities but not at the community level. In the first year of the study in the intervention district (Alamata), early clinical diagnosis of malaria and deployment of AL was conducted by 33 community health workers (CHWs) as well as by health workers in health facilities. Volunteers were chosen to act as CHWs, and were trained in correct clinical diagnosis of malaria, administration of AL, and community education. In the second year of the study, CHWs were provided with RDTs to help ensure that AL was only given to patients with malaria due to *P. falciparum*, with the aims of minimizing the risk of resistance developing and improving cost effectiveness by countering excessive and unnecessary administration of AL.

**Figure 4:**
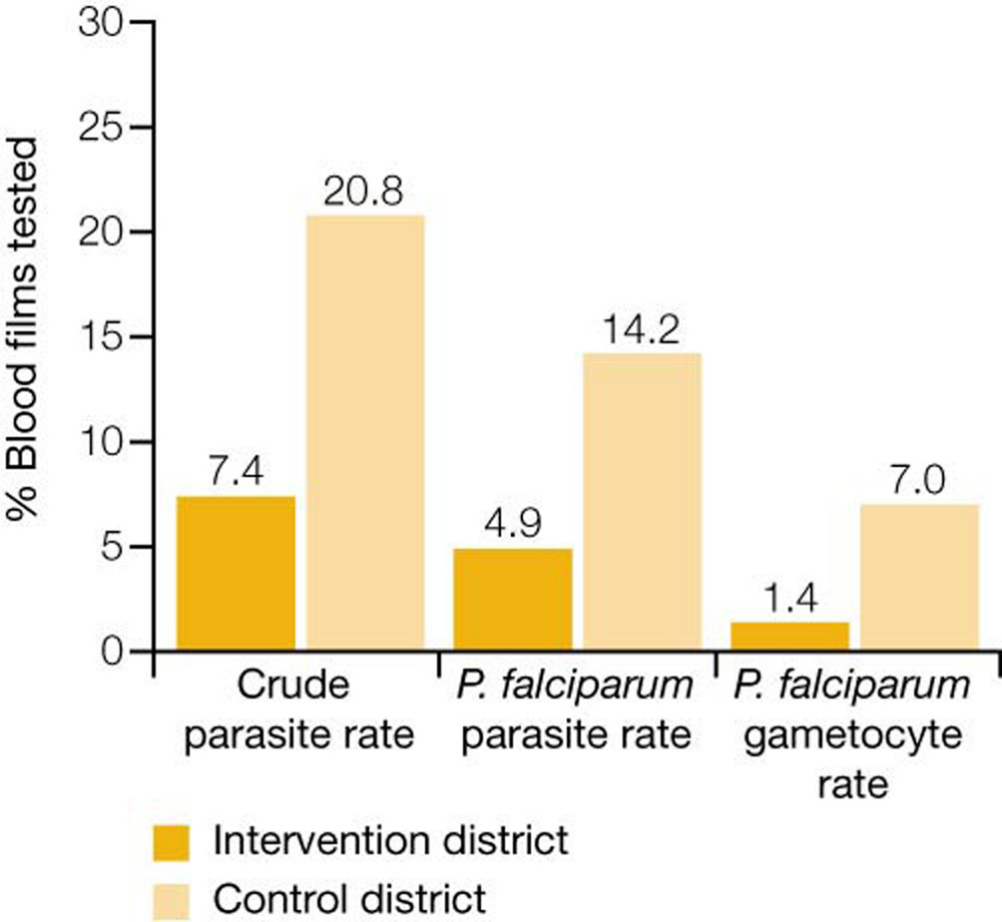
**Malaria parasite reservoir in the control and intervention districts of the Tigray study region**. Malaria parasite reservoir was three-fold lower in the intervention district during 2005 high-transmission season.

During the project, which involved over 200,000 individuals presenting with malaria-related symptoms, 58% of patients in the intervention district were treated for malaria by CHWs instead of at health facilities, thus reducing the malaria case load for general health services. A lower rate of *P. falciparum*-positive cases was reported in the intervention district compared with the control district (48% of 4,778 blood slides *versus *64% of 6,778 blood slides over the two-year study, respectively). During a major malaria epidemic from May-October 2005, the district in which the local volunteers were operating had approximately half the rate of malaria-related deaths compared with the district in which AL was available only in state health facilities (24 out of 991 deaths in the intervention district, *versus *53 out of 1106 deaths in the control district) [32]. Over the two-year period, the community-based deployment of AL significantly lowered the risk of malaria-specific mortality by approximately 37% (p = 0.013). Additionally, the malaria parasite reservoir was three-fold lower in the intervention district than in the control district during the 2005 high-transmission season (Figure 4). The use of RDTs by CHWs in the second year of the study allowed exclusion of non-*P. falciparum *malaria in 89.7% of cases, avoiding over-treatment with AL.

Community deployment of AL ensured that patients could be treated promptly, with access to effective therapy at all times. Overall, the intervention was associated with lower malaria transmission, a lower malaria case burden for health facilities, and reduced malaria morbidity and mortality.

## The use of ACT in Africa

Following the launch of the WHO 'Roll Back Malaria' campaign in 1998, few countries with endemic malaria had adopted ACT by 2000. The turning point came in January 2004, when an article was published in The Lancet [33], a constructive criticism of the lack of policy change written by prominent opinion leaders. A more rapid adoption of ACT since the beginning of 2004 resulted in at least 40 malaria-endemic countries in sub-Saharan Africa rapidly scaling up malaria prevention and treatment and recommending the use of ACT as first-line treatment for uncomplicated falciparum malaria [34].

Now is a critical time to establish the impact and sustainability of malaria control in Africa. AL continues to be provided in malaria-endemic African countries by Novartis on a non-profit basis, and more than 250 million AL treatments have been supplied to developing countries to date. The effect on patients is significant; African communities carrying the highest burden of malaria are increasingly able to access effective artemisinin-based combination therapy using locally available delivery models. Access to effective treatment reduces the risk of malaria mortality.

## Conclusion

Artemether-lumefantrine has had a remarkable impact in South Africa, Zambia and Ethiopia. Similar benefits have been seen with other forms of ACT, particularly artesunate-mefloquine on the western border of Thailand [35] and artesunate-amodiaquine in Zanzibar [36]. These parallel findings indicate that the public health impact is a class effect of the artemisinin-based combinations, and can be achieved when high rates of coverage and adherence are accomplished and the partner drug is highly effective.

Malaria is a life-threatening but fully preventable and treatable disease. By scaling up malaria control measures, the health and economic impact of the disease in Africa can be significantly reduced [37]. The impact of ACT on the disease, the synergistic effect on other control strategies and the healthcare benefits are being realized.

## Competing interests

The authors would like to acknowledge that Novartis Pharma AG sponsored this supplement. However, none of the authors works for, or represents in any way, Novartis Pharma AG.

## Authors' contributions

All authors met International Committee of Medical Journal Editors criteria for authorship.
